# *Titin* Mutation Is Associated With Tumor Mutation Burden and Promotes Antitumor Immunity in Lung Squamous Cell Carcinoma

**DOI:** 10.3389/fcell.2021.761758

**Published:** 2021-10-21

**Authors:** Xiaona Xie, Yemeng Tang, Jueqi Sheng, Pingping Shu, Xiayan Zhu, Xueding Cai, Chengguang Zhao, Liangxing Wang, Xiaoying Huang

**Affiliations:** ^1^The First Affiliated Hospital, Wenzhou Medical University, Wenzhou, China; ^2^School of Pharmaceutical Sciences, Wenzhou Medical University, Wenzhou, China

**Keywords:** lung squamous cell carcinoma, tumor mutation burden, immunotherapy, TCGA, ICGC

## Abstract

Lung squamous cell carcinoma (LUSC) is a leading cause of mobidity and mortality worldwide. Recently, there was a shift in the treatment pattern of immune therapy in LUSC patients; merely a small number of patients with non-small cell lung cancer (NSCLC) at advanced stages respond well to immune checkpoint blockade (ICB) therapy, and tumor mutation burden (TMB) is a valuable independent indicator of response to immune therapy. However, specific gene mutations and their relationship with TMB and tumor-infiltrating immunocytes in LUSC are still unclear. In the present paper, our team analyzed the somatically mutated genes from the ICGC (International Cancer Genome Consortium) and TCGA (The Cancer Genome Atlas) datasets and discovered that 15 frequent gene mutations occurred in both cohorts, including *ZFHX4*, *MUC16*, *FLG*, *TP53*, *LRP1B*, *TTN*, *SYNE1*, *RYR2*, *CSMD3*, *USH2A*, *MUC17*, *DNAH5*, *FAM135B*, *COL11A1*, and *RYR3.* Interestingly, only mutated *TTN* was related to higher TMB and prognostic outcomes among the 15 mutated genes. Moreover, according to the CIBERSORT algorithm, we revealed that *TTN* mutation enhanced the antitumor immune response. In conclusion, *TTN* may have important clinical implications for relevant immune therapy of lung squamous carcinoma.

## Introduction

Lung squamous cell carcinoma (LUSC) is one of the most commonly seen histological subtypes of non-small-cell lung cancer (NSCLC), the remaining and a global leading cause of mortality (Perez-Moreno et al., 2012; Chen et al., 2014; Gandara et al., 2015). Over the past few decades, there have been limited advances in lung squamous cell carcinoma therapy due to deficient mutation targets, and the chemical therapy based on platinum is still a normative therapy for advanced lung squamous cell carcinoma (Langer et al., 2016), due to a lack of therapeutic. Recently, immune checkpoint inhibitors (ICIs), targeting the programmed cell death-1 (PD-1) and programmed cell death ligand-1 (PD-L1) axis, have revolutionized cancer therapy. The majority of explorations discovered that the therapy exhibited more effectiveness in cases with positive PD-L1 compared with PD-L1-negative patients. Nevertheless, the PD-L1-negative group still demonstrated response rates of around 10%, implying that PD-L1 did not serve as a suitable biomarker for response (Herbst et al., 2014; Gettinger et al., 2015). Hence, exploring the determining factors on the molecular level of immunotherapy response is challenging in lung squamous cell carcinoma, and numerous researches are currently addressing this problem. Tumor mutation burden (TMB), an indicator of the entire mutation volume from cancer cells, is considered an underlying biomarker for the immunotherapeutic approach. Therefore, utilizing the expression of TMB or other clinical features as predictive factors to guide immunotherapies in real-world clinical practice has been remarkably highlighted. The latest study suggested that the greater TMB predicts a favorable outcome to PD-1/PDL1 inhibition in different cancers (Goodman et al., 2017). Higher TMB significantly predicts favorable outcomes to PD-1/PD-L1 blockade in NSCLC as well as small-cell lung tumors, implying that comprehensive genomic profiling may result in patient benefit. Whether TMB is related to the prognostic power of other biomarkers is still opaque.

The *TTN* gene is composed of 363 exons and displays the longest exon in the whole genome. Titin is implicated in conferring elasticity to sarcomere, sarcomere assembly, and mechanosensing (Roberts et al., 2015; Kellermayer et al., 2019), serving as a structural protein in striated muscles (Ciferri and Crumbliss, 2018). Mutated genes in this regard are associated with inherited hypertrophic cardiac muscle disease (Gerull et al., 2002), and autoantigen against titin has been identified in patients with the autoimmunity illness scleroderma (Ohyama et al., 2015). It has also been reported to be mutated frequently in many tumor types including breast cancer, lung squamous cell carcinoma, lung adenocarcinoma, and colon adenocarcinoma (Ceyhan-Birsoy et al., 2013). Lung squamous cell carcinoma acts as a genomically heterogeneous cancer with remarkable mutation ratios, and *TTN* acts as a frequent gene mutation in LUSC. However, the changes in *TTN* mutations and their relationship with TMB and immunocyte-infiltrating cancers in lung squamous cell carcinoma remain vague.

Our team initially detected somatically mutated genes in lung squamous cell carcinoma patients from America and *Asia via* TCGA and ICGC datasets. Afterward, we discovered the commonly mutated genes in the two cohorts and revealed in depth the relationship among the mutated genes, prognostic results, and TMB. Therefore, our purpose is to identify mutated genes using TCGA and ICGC lung squamous cell carcinoma specimens, and to further unveil the association of mutated genes with TMB and patient outcome and infiltrating immune cells.

## Materials and Methods

Transcriptome data and the data of somatically mutated genes of lung squamous cell carcinoma samples from the Amercian (*n* = 484) were obtained from the website of TCGA^1^ (March 30, 2021). Somatic mutation data for Asian lung squamous cell carcinoma samples (*n* = 170) were acquired from the website of ICGC^2^ (March 26, 2021). The clinical information of 484 LUSC samples was downloaded from TCGA. Data were extracted and organized in Perl so that it can be analyzed in R. As to the clinical information, only patients with lung squamous cell carcinoma with complete information were included, excluding any absent information such as survival status, age, gender, grade, and TNM information.

### Classification of Lung Squamous Cell Carcinoma Based on Tumor Mutation Burden

Tumor mutation burden was calculated as the total quantity of mutated bases per megabase, and only mutations that cause changes in amino acids were counted. The expression of TMB in each TCGA lung squamous cell carcinoma sample was calculated by the TMB formula (Chalmers et al., 2017).

### Bioinformatic Analysis

All bioinformatic analyses were performed by the R software (v4.0.1). Genes with the top 30 mutation frequencies in TGCA and IGGC databases were, respectively, extracted by Perl. The R package “GenVisR” was used to visualize the mutations of these genes (Mayakonda et al., 2018). These genes were intersected to obtain genes with high mutation frequency in both databases by the R package “venn.” The relationship between those intersection mutated genes and TMB was assessed and visualized *via* the R package “ggpubr.” GSEA analysis was performed using *TTN* mutation and expression matrix data in the GSEA software (v4.1.0) (Subramanian et al., 2005). Normalized enrichment score (NES) was calculated by setting the permutation values to 1,000, and the FDR *p*-value < 0.05 was adopted to determine evident enrichment pathways. CIBERSORT is a computational method for assessing the proportion of 22 immunocyte immune cells in tumor tissue based on transcriptome data (Newman et al., 2015). Matrix data of immune cell proportion for each tumor sample were obtained using CIBERSORT deconvolution algorithm setting the filter condition to *p* < 0.05. The matrix data visualization was performed by R package “corrplot.” TCGA samples were assigned to the wild group and the mutation group based on *TTN* status. Difference analysis of immunocyte infiltration within the two groups was performed by R package “limma” and visualized by R package “vioplot.”

### Statistical Analysis

Statistical analysis was implemented *via* R (v4.0.1). Survival curves were analyzed *via* Kaplan–Meier survival analysis and evaluated using the log-rank test. Identification of prognosis risk factors was done by performing survival analysis of the clinical characteristics of patients, including age, gender, grade, and TNM classification by univariate and multivariate Cox regression analyses. The correlation between mutant genes and TMB was studied by the Mann–Whitney *U*-test. For all comparisons, a two-tailed *p* < 0.05 had statistics-related significance.

## Results

### Somatic Mutation Characteristics in Lung Squamous Cell Carcinoma

**FIGURE 1:**
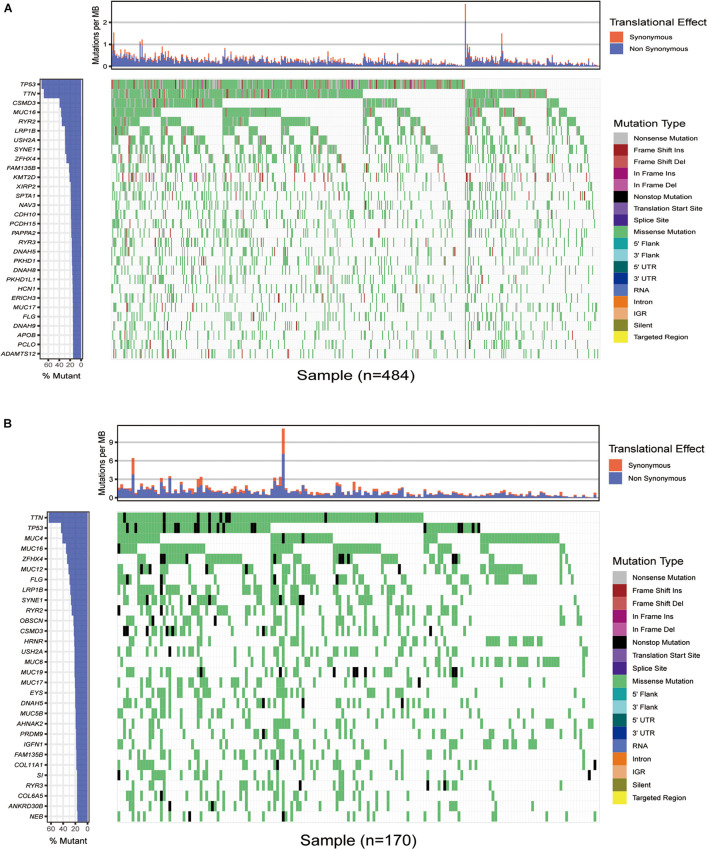
Overview of frequently mutated genes in lung squamous cell carcinoma. **(A)** Waterfall plot shows the frequent gene mutations in lung squamous cell carcinoma from The Cancer Genome Atlas (TCGA) database. The left panel presents the frequency of gene mutation according to which genes are sequenced. The right panel displays diverse mutations. **(B)** Waterfall plot displaying the frequent gene mutations in lung squamous cell carcinoma from the International Cancer Genome Consortium (ICGC) cohort. The left panel exhibits the genes sequenced *via* the frequency of gene mutation. The right one displays diverse mutations.

We first downloaded the mutation data of 484 American lung squamous cell carcinoma samples from TCGA, and the cumulative mutation frequency in each gene was counted and sorted in decreasing order. The top 30 frequently mutated genes with high mutation frequency and pattern of somatic mutation for the top 30 genes are illustrated in Figure 1A. The top 30 mutated genes were *TP53*, *TTN*, *CSMD3*, *MUC16*, *RYR2*, *LRP1B*, *USH2A*, *SYNE1*, *ZFHX4*, *FAM135B*, *KMT2D*, *XIRP2*, *SPTA1*, *CDH10*, *NAV3*, *CDH10*, *PCDH15*, *PAPPA2*, *RYR3*, *DNAH5*, *PKHD1*, *DNAH8*, *PKHD1L1*, *HCN1*, *ERICH3*, *MUC17*, *FLG*, *DNAH9*, *APOB*, *PCLO*, and *ADAMTS12*. Similarly, the top 30 genetic mutations were also identified in Asian patients from the ICGC database. As shown in Figure 1B, missense mutation occurred commonly in Asian patients, and *TTN*, *TP53*, *MUC4*, *MUC16*, *ZFHX4*, *MUC12*, *FLG*, *LRP1B*, *SYNE1*, *RYR2*, *OBSCN*, *CSMD3*, *HRNR*, *USH2A*, *MUC6*, *MUC19*, *MUC17*, *EYS*, *DNAH5*, *MUC5B*, *AHNAK2*, *PRDM9*, *IGFN1*, *FAM135B*, *COL11A1*, *SI*, *RYR3*, *COL6A5*, *ANKRD30B*, and *NEB* had the top 30 mutation frequency among Asian patients.

### Gene Mutations Associated With Tumor Mutation Burden

**FIGURE 2:**
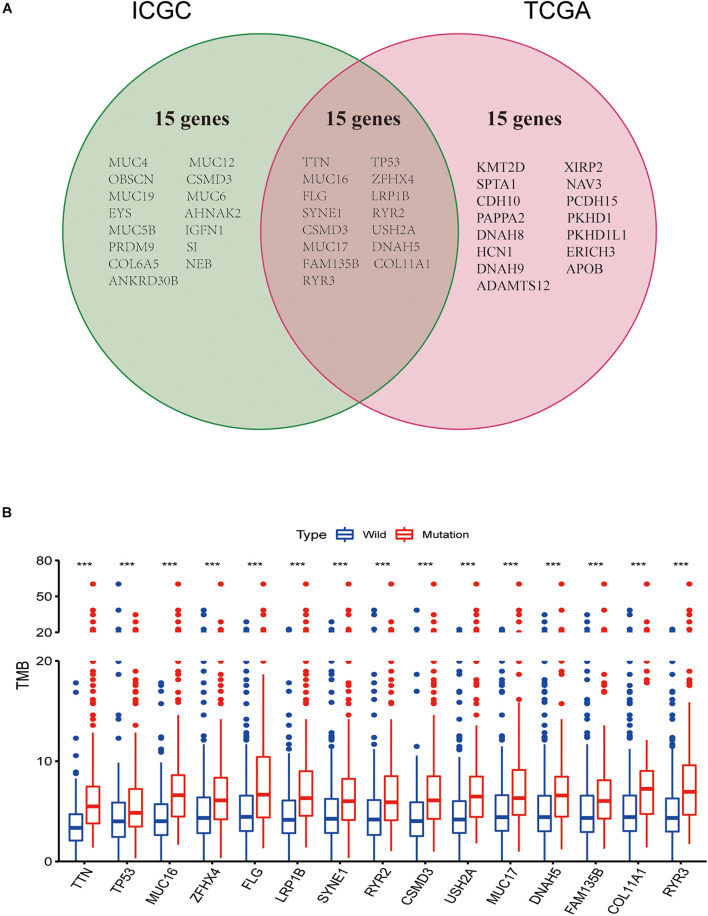
Gene mutations are associated with tumor mutation burden (TMB). **(A)** Venn diagram shows 15 frequent gene mutations of the TCGA and ICGC cohorts. **(B)** Fifteen genes with high mutation frequency are associated with a higher TMB. ***p < 0.001.

Intriguingly, we discovered common mutant genes in both TCGA and ICGC databases. As shown in Figure 2A, the intersection genes with high mutations were *TTN*, *TP53*, *MUC16*, *ZFHX4*, *FLG*, *LRP1B*, *SYNE1*, *RYR2*, *CSMD3*, *USH2A*, *MUC17*, *DNAH5*, *FAM135B*, *COL11A1*, and *RYR3.* To further investigate whether these 15 commonly mutated genes were associated with TMB, lung squamous cell carcinoma patients from the TCGA cohort were classified into wild and mutated groups based on the 15 mutant gene statuses. In addition, the TMB score in lung squamous cell carcinoma patients varies from 0.02 to 60 per Mb with an average value of 4.68 per Mb. Combining the analysis of the data of gene mutation matrix and TMB expression matrix, we found that the TMB value in the mutation group of all the other 15 genes was significantly changed compared with that of the wild group (Figure 2B).

### *TTN* Mutation Associated With Prognosis

**FIGURE 3:**
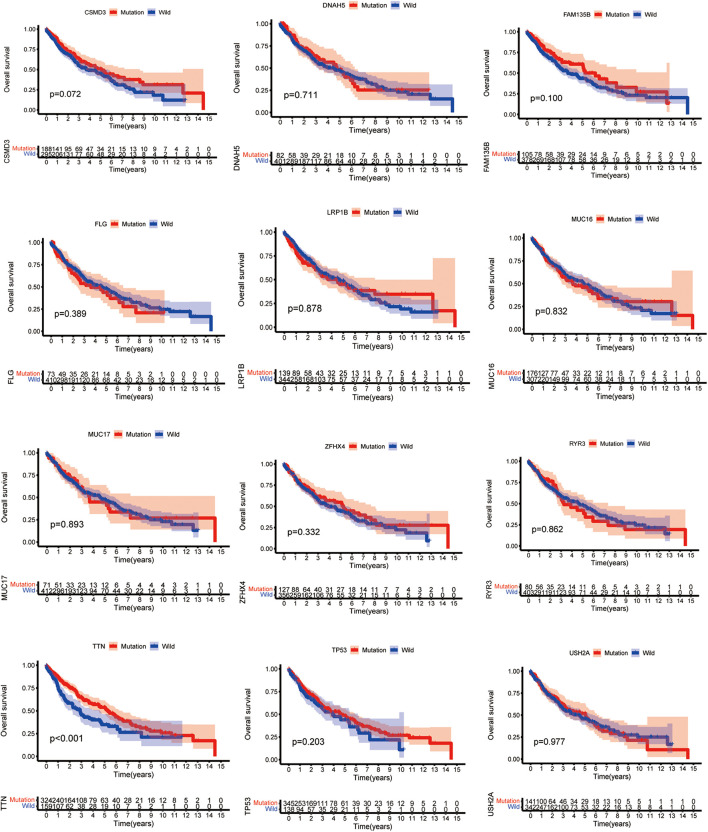
*TTN* mutation is associated with clinical prognosis. The Kaplan–Meier survival study was adopted to determine survival curves reflecting the relationship between mutant genes and prognostic results. The *p*-value is shown in every illustration.

**FIGURE 4:**
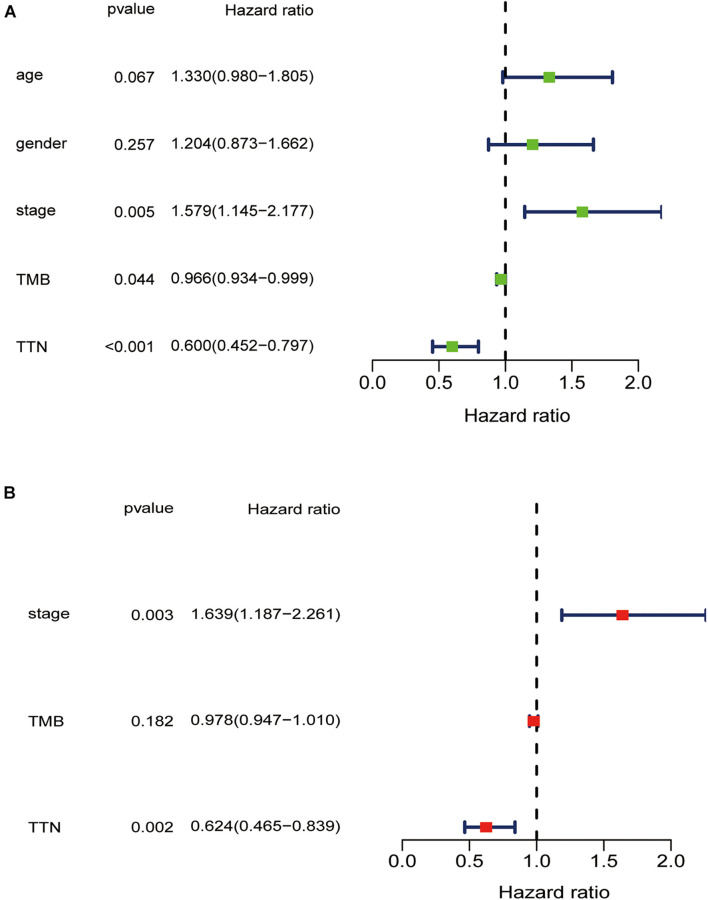
Univariate **(A)** and multivariate **(B)** overall survival analysis of lung squamous cell carcinoma patients *via* the Cox proportional hazards model.

Higher TMB significantly predicts a desirable result of PD-1/PD-L1 blockade both in NSCLC and SCLC, implying that comprehensive genomic profiling analysis might lead to patient benefit (Hellmann et al., 2018; Reck et al., 2019; Ricciuti et al., 2019). Thus, considering the established association between 15 mutated genes and TMB, we speculate that these genes may be associated with clinical outcomes. For this purpose, patients from the TCGA database were assigned to the wild and mutation groups according to gene mutation status, and Kaplan–Meier analysis was conducted combined with the analysis of patient survival data. Our results demonstrated that only *TTN* mutation was associated with a positive prognosis (*p* = 0.0008) (Figure 3). Based on this finding, we aimed to further identify whether *TTN* mutation is the independent prognostic factor for lung squamous cell carcinoma using Cox regression analysis. As shown in Figure 4, with correction for common clinical information and TMB score, *TTN* mutation remained significantly associated with the overall survival of patients.

### Enrichment Pathway Analysis of *TTN* Mutation

**FIGURE 5:**
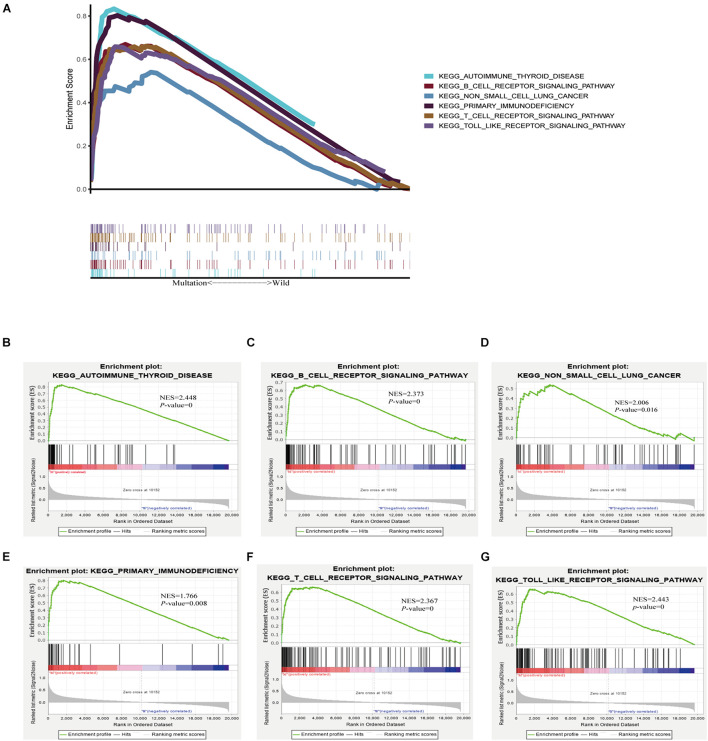
*TTN* mutation is associated with immune-related pathways. Gene set enrichment analysis was performed with the TCGA. **(A)** Multiple gene enrichment plot shows that a series of gene sets are enriched in the *TTN*-mutant group. Gene enrichment plots display that a series of immune-related gene sets, including **(B)** autoimmune thyroid disease, **(C)** B-cell receptor signaling pathway, **(D)** non-small-cell lung cancer, **(E)** primary immunodeficiency, **(F)** T-cell preceptor signaling pathway, **(G)** Toll-like receptor singling pathway, are enriched in the *TTN*-mutant group. NES, normalized enrichment score. The *p*-value is shown in each plot.

As TMB is reported to be a biomarker for immunotherapy, and *TTN* mutation was associated with an increased TMB, we further investigated the relation between *TTN* mutation and immune response. GSEA performed with TCGA revealed that autoimmune thyroid disease, B-cell receptor signaling pathway, non-small-cell lung cancer, primary immunodeficiency, T-cell preceptor signaling pathway, and Toll-like receptor singling pathway were significantly enriched in samples with *TTN* mutation (Figures 5A–G). These findings indicate that samples with *TTN* mutation upregulated signaling pathways involved in the immune system.

### Tumor-Infiltrating Immune Cells Associated With *TTN* Mutation in Lung Squamous Cell Carcinoma

**FIGURE 6:**
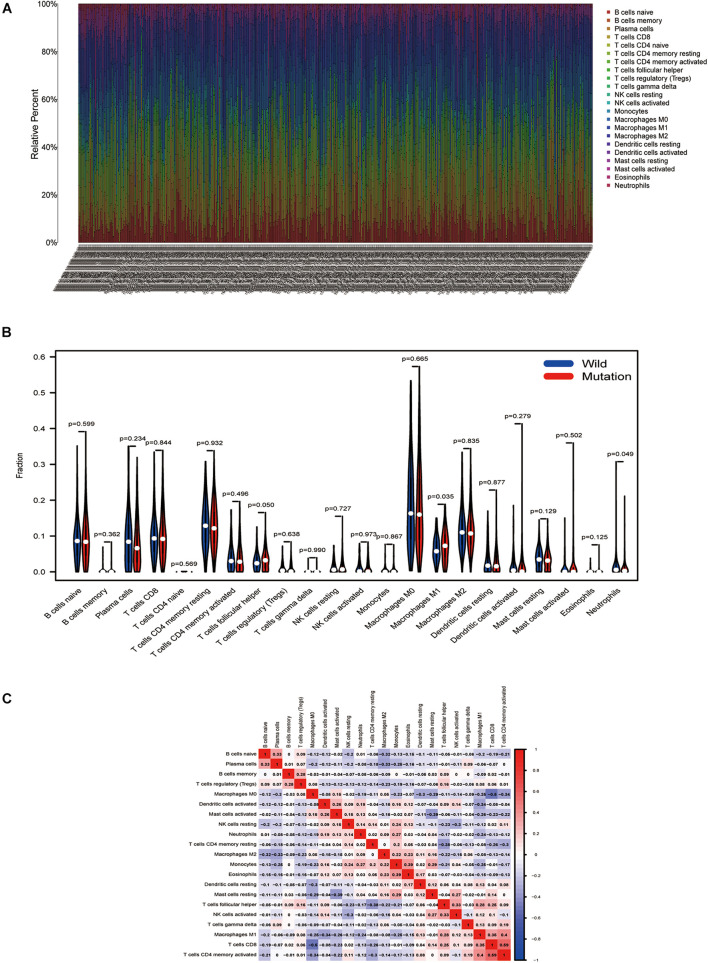
*TTN* mutation is correlated with tumor-infiltrating immune cells. **(A)** The stacked bar chart shows the distribution of 22 immunocytes in each sample. **(B)** Violin plot displaying the differentiated immunocyte infiltration between the mutated *TTN* and the wild-type *TTN* group. Blue refers to the wild-type *TTN* group, and red reflects the *TTN*-mutant group. The *p*-value is shown in the figure. **(C)** Correlation matrix of immunocyte ratios. The red color represents positive correlation, and the blue color represents negative association.

Using the CIBERSORT deconvolution algorithm, we first calculated the proportion of 22 immunocytes for each sample in tumor tissue (Figure 6A). The results revealed that the number of infiltrating immune cells changes greatly in different samples; macrophage M1 was more enriched in the TTN mutation group; however, neutrophils were enriched in the wild group (Figure 6B). Finally, correlation analysis revealed that macrophage M1 exhibited the most potent affirmative association with activated memory CD4 T cells and also positively associated with CD8 T cells, while they were reversely associated with stimulated dendritic cells (Figure 6C). Moreover, neutrophils had the strongest positive correlation with monocytes and had the strongest negative correlation with macrophage M1 (Figure 6C).

## Discussion

In the past decades, tumor-associated neoantigens, derived from non-synonymous somatic mutations, have been identified as the prime targets of cytotoxic T lymphocytes within cancer microenvironments (Chen et al., 2019). Those cancer cell-identifying T cells could be enhanced *via* the administration of ICB agents, either by reinvigorating the depleted cytolysis or *via* consuming various types of immune regulation cells (Park et al., 2019). For the time being, no consentaneous agreement exists on the way to obtain effective neoantigens from substantial genome data. We, respectively, described the somatically mutated genes of 484 LUSC specimens in the United States from TCGA and the LUSC specimens of 170 Asian patients from the ICGC dataset. Consequently, mutated *TTN* was related to higher TMB and beneficial clinical outcomes. Meanwhile, samples with *TTN* mutation positively correlated with signaling pathways implicated in immune response. Tumor-infiltrating immune cell results demonstrated that *TTN* mutant samples presented a higher infiltration proportion of macrophage M1, and less infiltrated in neutrophils, which supported the previous findings that such immune cells and pathways play predominant roles in the tumor microenvironment and promote immune response (Hsu et al., 2018; Zuazo et al., 2019). Therefore, our results reveal that mutated *TTN* might be a convenient prediction for ICB immune treatment in lung squamous cell carcinoma patients.

Tumor mutation burden, referring to the overall mutation volume per coding area in the cancer genome, is high in cancer specimens and has become an underlying marker in tumor immune treatment (Goodman et al., 2017; Yarchoan et al., 2017). TMB reflects the accumulation of somatically mutated genes in cancers, and the TMB facilitates the effectiveness of more neoantigens, which may induce an immune response depending on T cells. The research revealed that the higher TMB predicts a favorable result of PD-1/PDL1 suppression in different cancers (Goodman et al., 2017). Higher TMB notably predicts favorable outcomes to PD-1/PD-L1 blockade in both NSCLC and SCLC, implying that comprehensive analysis might lead to patient benefit. In our study, nonsynonymous mutated genes in Titin associate with higher TMB, which is related to desirable clinical results of patients with *TTN* mutations. *TTN* has also been reported to be mutated frequently in many types of tumors such as breast cancer, lung squamous cell carcinoma, lung adenocarcinoma, and colon adenocarcinoma (Ceyhan-Birsoy et al., 2013). *TTN* is a frequently mutated gene in lung squamous cell carcinoma, and autoantibodies against titin are identified in patients with the autoimmune disease scleroderma (Ohyama et al., 2015). Hence, we speculated that *TTN* mutation with a high TMB in lung squamous cell carcinoma might drive the immune system to fight against tumor cells.

Interestingly, *TTN* mutant samples presented a higher infiltration proportion of macrophage M1 and less infiltrated in neutrophils. Previous research evidenced that macrophages were vital in the immune system, and differentiated macrophages mark a pivotal factor for immunity response (Ivanova and Orekhov, 2016). Macrophage M1 is a primary cell subtype of tumor-associated macrophages (TAMs), and it has an inflammation-promoting effect, and immunogenic and antitumor properties, which are an important part of the tumor microenvironment (Yang and Zhang, 2017; Singhal et al., 2019). Our findings reveal that mutated *TTN* was related to macrophage M1. Macrophage M1 exhibited the most potent positive correlation with activated memory CD4 T cells and is positively related to CD8 T cells as well, which confirmed the previous evidence that antitumor immune response was associated with these immune cells. Therefore, we assumed that *TTN* mutation might positively regulate macrophages M1, CD4, and CD8 T cells in lung squamous cell carcinoma. Thus, our results demonstrated that the changed tumor-infiltrating immune cells induced by *TTN* contribute to the antitumor immunity of lung squamous cell carcinoma.

The main limitation in our study is that the ICGC database lacks corresponding clinical data of China lung squamous cell carcinoma, so we cannot verify whether *TTN* mutation is associated with the prognosis of lung squamous cell carcinoma patients in China and whether it can give rise to the same immune response. Even if *TTN* is also frequently mutated in Chinese lung squamous cell carcinoma samples, the effect may be somewhat heterogeneous between different ethnic groups. Consequently, the relationship between mutant *TTN* and prognostic results, such as immunocyte infiltration and signaling pathways, demands more verification in lung squamous cell carcinoma specimens from China.

## Conclusion

In conclusion, this work demonstrated that mutated *TTN* was frequently identified in lung squamous cell carcinoma, and mutated *TTN* was related to higher TMB and indicated prognostic result. Additionally, mutated *TTN* evoked an antitumor immune response. Our discoveries unveil a novel gene, the mutation of which can act as a biomarker to predict immune response.

## Data Availability Statement

The datasets presented in this study can be found in online repositories. The names of the repository/repositories and accession number(s) can be found in the article/supplementary material.

## Author Contributions

XX and YT carried out most of the experiments. JS analyzed the data and prepared the figures. XC and XZ conceived the idea and designed the research. XX and YT wrote the manuscript. All authors read and approved the final version of the manuscript.

## Conflict of Interest

The authors declare that the research was conducted in the absence of any commercial or financial relationships that could be construed as a potential conflict of interest.

## Publisher’s Note

All claims expressed in this article are solely those of the authors and do not necessarily represent those of their affiliated organizations, or those of the publisher, the editors and the reviewers. Any product that may be evaluated in this article, or claim that may be made by its manufacturer, is not guaranteed or endorsed by the publisher.

## Footnotes

1
http://portal.gdc.cancer.gov/projects

2
http://dcc.icgc.org/releases/current/Projects

## Abbreviations

LUAD: lung adenocarcinoma
LUSC: lung squamous cell carcinoma
NSCLC: non-small-cell lung cancer
TCGA: The Cancer Genome Atlas
ICGC: International Cancer Genome Consortium.
